# Single center experience: clinical features of children with atopic dermatitis

**DOI:** 10.1186/2045-7022-1-S1-P23

**Published:** 2011-08-12

**Authors:** Aysegul Akan, Mustafa Erkocoglu, Aysenur Kaya, Dilek Azkur, Celal Ozcan, Muge Toyran, Ersoy Civelek, Can Naci Kocabas

**Affiliations:** 1 Ankara Hematology Onkology Children’s Training and Research Hospital, Pediatric Allergy, Ankara, Turkey

## Background

Atopic dermatitis (AD) is a common chronic skin disease that can vary in degree of severit. The factors affecting the severity of AD are not known well. In this study, the clinical features of patients with different disase severity were compared if there is any significant difference between patients with mild, moderate and severe atopic dermatitis.

## Methods

The patients with AD admitted to outpatient clinic of pediatric allergy department from 1 January 2010 to 30 June 2010 were enrolled to the study. The disease severity and risk factors of the patients were assesed according to the SCORAD index. Skin prick test (SPT) for common food and inhalent allergens were performed to all patients with atopic dermatitis. The food specific IgE blood tests (Phadia ImmunoCap Uppsale, Sweden) were performed to only selected patients who had symptoms on the basis of clinical history.

## Results

Total of 134 patients were evaluated with average age of 31.4±3.5 months, 58.2% of patients were male. The mean age of the start of symptoms and the duration of breast-feeding were 8.2±1.2 and 8.8±0.5 months, respectively. The frequency of taking cow's milk in the first year of life was 29.1% (n=39). The 29.9% of patients had family history of atopy. Peripheral blood eosinophil ratio was 3.3% (2.0 -5.0) (median, interquartle range 25-75%) and total blood IgE level was 32.6 (10.4-100.0) kU/L. The 70.1% of patients was higher than the normal range for age. The SCORAD index was 36.1 (26.4-45.6). The frequency of mild, moderate and severe atopic dermatitis among the patients were 19.4, 62.7 and 17.9%, respectively. The 42.5% of patients were sensitized to the allergens tested. The most frequent sensitized allergens were egg white (26.9%), cow’s milk (10.4%), wheat (6.0%). Among the patients with different severity, there were no significant difference according to gender, the status of atopy, age, the beginning age of symptoms, the percent of peripheral blood eosinophil, family history of atopy, serum IgE level.

## Conclusions

The most common form was moderate AD. There were no differences of clinical features among mild, moderate and severe atopic dermatitis.

